# Graphene and Vanadium Dioxide-Based Terahertz Absorber with Switchable Multifunctionality for Band Selection Applications

**DOI:** 10.3390/nano14141200

**Published:** 2024-07-15

**Authors:** Yan Liu, Lingxi Hu, Ming Liu

**Affiliations:** 1 School of Microelectronics, Shenzhen Institute of Information Technology, Shenzhen 518172, China; 2021000310@sziit.edu.cn (Y.L.); lium@sziit.edu.cn (M.L.); 2 Digital and Intelligent Agriculture Research Institute, School of Information Engineering, Huzhou University, Huzhou 313000, China

**Keywords:** terahertz absorber, switchable multifunctionality, band selector, excellent absorptance

## Abstract

This study proposes a multifunctional absorber in the terahertz (THz) regime based on vanadium dioxide (VO_2_) and graphene with either–or band selector applications, which can be realized by electrically and thermally controlling the Fermi energy level of graphene and vanadium dioxide, respectively. The broadband absorption can be achieved with absorptance exceeding 90%, when the VO_2_ film is in the metallic phase and the Fermi energy levels of the upper and lower graphene layers are simultaneously set to 0.6 and 0 eV, respectively. The double narrowband can be realized when the VO_2_ film is in the insulating phase and the Fermi energy levels in upper and lower graphene layers are set as 0 and 0.8 eV, respectively. By flexibly shifting between the broadband and the double narrowband, the proposed absorber can be used as an either–or band selector, corresponding optional bandwidth from 2.05 to 2.35 THz, and 3.25 to 3.6 THz. Furthermore, single narrowband absorption can be achieved by setting the conductivity of the VO_2_ film to appropriate values. The proposed absorber can be used in the THz regime in applications such as multifunctional devices, switches, cloaking objects, and band selectors.

## 1. Introduction

Metamaterial, a kind of artificial material with unusual electromagnetic properties [1,2], has acquired much attention for its widely applications in perfect lens [3], thermal radiation [4], photodetectors [5], optical polarizers [6], and perfect absorbers [7]. Terahertz (THz) radiation ranging from 0.1 to 10 THz has considerable potential in future applications such as wireless communication [8,9], medical imaging [10], and nondestructive testing [11]. In recent years, metamaterial-based THz absorbers [12,13] have been extensively studied, focusing on four aspects: structural optimization, performance improvement, frequency response, and functional expansion. To meet the need of miniaturization and integration, the thickness of the absorber has been reduced [14] for optimizing its structure, which is also a significant research direction. The investigations of performance improvement mainly focus on expanding the bandwidth of the broadband absorption [15], and reducing the polarization insensitivity [16]. The frequency response of narrowband [17], broadband [18,19], single-band, and multiband [20,21] absorbers has been widely investigated. Furthermore, the functionalities of absorbers have been expanded to various applications, such as resonators [22], reflectors [23], and modulators [24].

The metamaterial-based multifunctional THz devices have recently garnered considerable interest in recent years. Multifunctional THz devices with reconfigurable characteristics are usually integrated with functional materials such as graphene [25,26,27], phase-change materials [28,29], doped semiconductors [30,31], and ferroelectrics [32,33]. Graphene is widely used among these materials owing to its excellent mechanical, electrical, magnetic, and thermal properties [34]. The conductivity of graphene can be dynamically adjusted by external voltages [35] attributed to its excellent electrical properties. Moreover, the phase transition of vanadium dioxide (VO_2_) can be triggered by electrical [36], thermal [37], and optical excitation [38]. For thermal control, the VO_2_ film shows a reversible transition from a metallic state to an insulating state at ~341 K.

Herein, an absorber composed of squared graphene metamaterials and a VO_2_ film is developed, which could achieve switchable multifunctionality with either–or band selector applications in the THz regime. The proposed absorber could flexibly switch its broadband absorption to dual narrowband absorption based on the insulator-to-metal phase transition of VO_2_ and electrically tunable property of graphene. When the VO_2_ film was in the metallic phase, the Fermi energy levels of the upper and lower graphene layers were simultaneously set to 0.6 and 0 eV to achieve broadband absorption. When the VO_2_ film was in the insulating phase, the Fermi energy levels in the upper and lower layers were set to 0 and 0.8 eV, respectively, for the absorber to achieve dual narrowband absorption. Owing to this flexible shift in the absorption band, the absorber can be used as an either–or band selector with optional bandwidth from 2.05 to 2.35 THz, and 3.25 to 3.6 THz. Furthermore, the absorber achieved single narrowband absorption when the VO_2_ conductivity was set to an appropriate value. Additionally, the influence of various geometrical parameters on the absorption spectra was investigated, and field analyses were performed to reveal the absorption mechanism. Generally, the proposed absorber promises multifunctionality in the THz regime, including broadband absorption, dual narrowband absorption, band selection, and single narrowband absorption.

## 2. Materials and Methods

**Figure 1:**
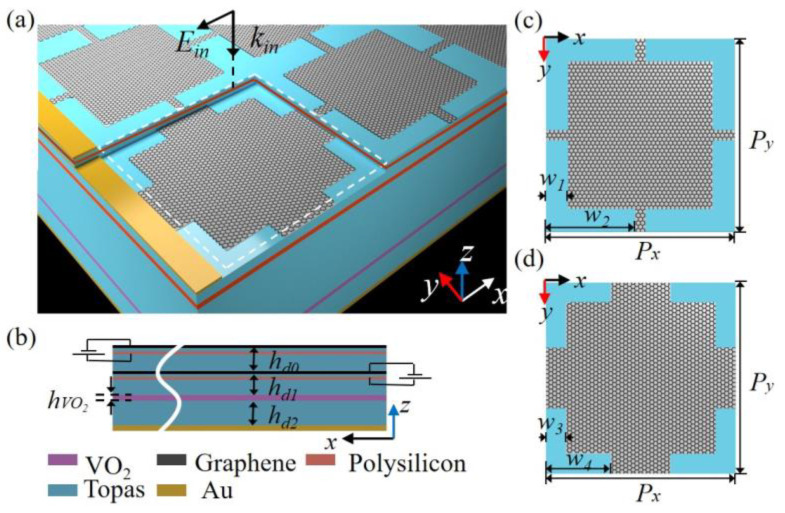
(**a**) Schematic of the multifunctional absorber with the polarization configuration of incident light. (**b**) Side view of the multifunctional absorber. (**c**) Top view of the unit cell for the upper square graphene layer and (**d**) lower square graphene layer.

Figure 1a schematizes the multifunctional absorber for wide-band absorption, narrowband absorption, and either–or band selection. The proposed structure is composed of double squared graphene layers with different dimensions, a 2 μm thick VO_2_ layer, and a gold (Au) plate as the bottom reflector. The layers are separated by an insulating material, Topas (polyethylene cyclic olefin copolymer) [39]. The Fermi energy of the squared graphene layers was controlled using gold ring films as the upper electrode and an extremely thin polysilicon layer as the lower electrode; the ultra-thin lower electrode slightly affects the absorption [40]. A THz wave with *x* polarization was emitted along the z-axis. Figure 1b shows the side view of the multifunctional absorber. Figure 1c,d show the unit cells of the upper and lower graphene layers, respectively. Table 1 lists the detailed dimensions of the proposed absorber.

The relative permittivity of Topas is 2.35. The conductivity of gold can be described by the Drude model with a plasma frequency *ω*_p_ of 1.36 × 10^16^ rad/s and scattering rate Γ of 3.33 × 10^13^ rad/s. The proposed absorber is numerically simulated using the finite-element solver COMSOL Multiphysics. The unit cell in *x* and *y* directions is treated with the periodic boundary condition, and the graphene layer is modeled as a surface current [41].

The CVD-grown graphene layer is transferred onto the multilayer substrate by a transfer technique using polymethylmethacrylate (PMMA) supporting layers, and is subsequently patterned by photolithography and oxygen plasma etching. The source-drain contacts are deposited on top of lower-layer graphene using an electron-beam lithography step. The upper-layer graphene with source-drain contacts is prepared on the upper Topas layer in the same way. Based on the Kubo formula, the complex surface conductivity of graphene was determined as follows [42]:

(1) $$\sigma_{g}\left(w,\tau ,\mu_{c}\right)=\sigma_{\mathrm{int}ra}\left(w,\tau ,\mu_{c}\right)+\sigma_{\mathrm{int}ter}\left(w,\tau ,\mu_{c}\right),$$

(2) $$\sigma_{\mathrm{inter}}\left(w,\tau ,\mu_{c}\right)\approx \frac{{je}^{2}}{4\pi \hbar }\ln\left[\frac{2\left|\mu_{c}\right|-\left(\omega +j/\pi \right)\hbar }{2\left|\mu_{c}\right|+\left(\omega +j/\pi \right)\hbar }\right],$$

(3) $$\sigma_{\mathrm{intra}}\left(w,\tau ,\mu_{c}\right)\approx j\frac{e^{2}k_{B}T}{\pi \hbar^{2}\left(\omega^{2}+j\tau^{-1}\right)}\left[\frac{\mu_{c}}{k_{B}T}+2\ln\left(\exp\left(-\frac{\mu_{c}}{k_{B}T}\right)+1\right)\right],$$

where *T*, *ω*, *τ*, *k*_B_, *μ*_c_, and *ħ* ≈ 1.055 × 10^−34^ J·s denote the temperature in Kelvin, incident angular frequency, relaxation time, Boltzmann constant, chemical potential, and reduced Planck constant, respectively. When *k*_B_ < *μ*_c_, *μ*_c_ is equal to the Fermi energy level *E*_f_. The relaxation time *τ* can be described as *τ* = *μE_*f*_e*^−1^*υ_F_*^−2^ [43], and the carrier mobility *μ* is 1500 cm^2^V^−1^s^−1^.

**Figure 2:**
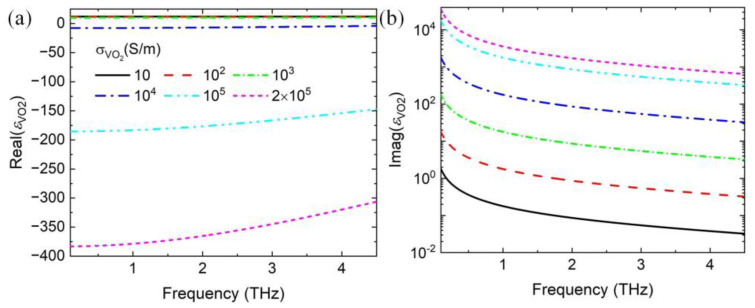
Real part (**a**) and imaginary part (**b**) of the complex relative dielectric permittivity of VO_2_ with varying conductivity.

The complex relative dielectric permittivity of VO_2_ can be expressed as follows [44]:

(4) $$\varepsilon \left(\omega \right)=\varepsilon_{\infty }-\frac{\omega_{p}^{2}\left(\sigma \right)}{\omega^{2}+i\gamma \omega },$$

where *ε*_∞_ = 12 is the dielectric permittivity at the infinite frequency, *γ* = 5.75 × 10^13^ rad/s is the collision frequency. *ω_p_*(σ) is the plasma frequency depending on conductivity, which can be approximately expressed as $\omega_{p}^{2}\left(\sigma \right)=\sigma^{2}\omega_{p}\left(\sigma_{0}\right)/\sigma_{0}^{2}$ with *σ*_0_ = 3 × 10^5^ S/m and *ω_p_*(*σ*_0_) = 1.4 × 10^15^ rad/s. During numerical simulation, the conductivities of VO_2_ film are set as 0 and 2 × 10^5^ S/m for the insulating and metallic phases, respectively. Figure 2 shows the real and imaginary parts of the permittivity of VO_2_ as a function of frequency with varying conductivity. As the conductivity increased, the real part of permittivity changes from more than zero to less than zero, and the imaginary part increases.

## 3. Results and Discussion

**Figure 3:**
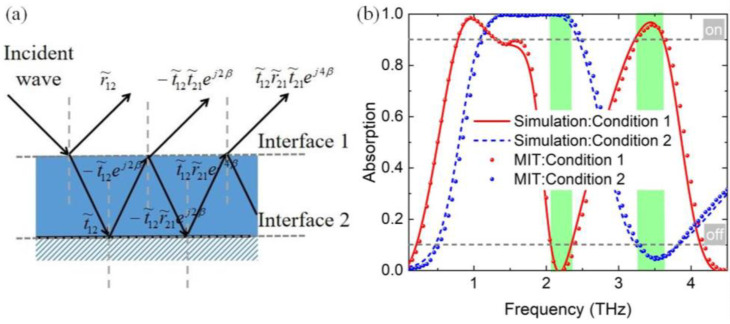
(**a**) Schematic of incident wave interferences between interfaces. (**b**) Absorption spectra of the multifunctional absorber in different states. The green shadow areas indicate the optional bandwidths.

The absorption spectra of the proposed absorber can be well explained by the multiple interference theory (MIT), which well validated the results of the simulations. When *E*_f1_ = 0.6 eV, *E*_f2_ = 0 eV, and VO_2_ in a metallic state (Condition 1), the upper graphene layer and the VO_2_ film can be used as a partial reflector and a total reflector, respectively. As shown in Figure 3a, the incident waves are reflected and refracted several times. Since the reflection coefficient of the VO_2_ film (in a metallic state) is −1, the reflection coefficient of the proposed absorber can be calculated as follows [45,46]:

(5) $$\tilde{r}={\tilde{r}}_{12}-\frac{{\tilde{t}}_{12}{\tilde{t}}_{21}e^{i2\tilde{\beta }}}{1+{\tilde{r}}_{21}e^{i2\tilde{\beta }}},$$

where $\tilde{\beta }=\sqrt{{\tilde{\varepsilon }}_{\mathrm{spacer}}}k_{0}d$ denotes the phase constant, *k*_0_ is the free space wavenumber, and *d* is the propagation length of the incident wave inside the proposed absorber. ${\tilde{r}}_{12}$ and ${\tilde{r}}_{21}$ are the reflection coefficients, ${\tilde{t}}_{12}$ and ${\tilde{t}}_{21}$ are the transmission coefficients. Both the reflection and transmission coefficients at the interface with graphene metamaterials can be obtained from simulations using the unit cell without the reflection structure. Since the transmission of the proposed absorber is zero, the absorptance can be then obtained by: $A=1-{\left|\tilde{r}\right|}^{2}$. Similar to Condition 1, the absorptance for the dual-narrowband can be obtained with *E*_f1_ = 0 eV, *E*_f2_ = 0.8 eV, and VO_2_ in the insulating state (Condition 2). It is clear that the simulated results (solid and dashed curves) are well consistent with the theoretical results calculated by MIT (spherical scatters), as shown in Figure 3b.

The proposed absorber achieves broadband absorption from 1.1 to 2.45 THz with absorptance exceeding 90% with Condition 1, and the dual-narrowband absorption can be obtained with Condition 2. Herein, the states “on” and “off” are assumed to be observed when the absorptances > 90% and <10%, respectively. Owing to the variations in the two absorption states, the absorber can be used as an either–or band selector (indicated by the green shadow areas), corresponding optional bandwidth from 2.05 to 2.35 THz, and 3.25 to 3.6 THz.

### 3.1. Broadband Absorption

**Figure 4:**
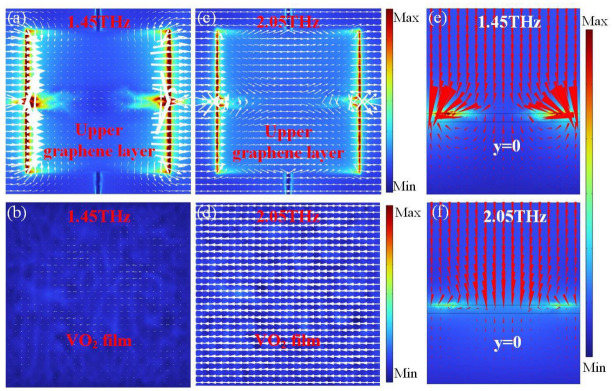
Distributions of the electric field amplitude |E| for (**a**,**c**) the upper graphene layer and (**b**,**d**) the VO_2_ film at the first and second resonant frequencies of 1.45 and 2.05 THz, respectively. Surface currents are marked with white arrows. Distributions of the power flow (red arrows) at the central cross-section of unit cell at (**e**) 1.45 THz and (**f**) 2.05 THz, respectively.

The distributions of electric field amplitude |E| at different resonant frequencies are analyzed to reveal the broadband absorption mechanism. The parameters of the structure are retained as Table 1, *E*_f1_ = 0.6 eV, *E*_f2_ = 0 eV, and VO_2_ in the metallic state, except for special notes. The induced currents are marked with white arrows in Figure 4. At the first resonant frequency of 1.45 THz, the electric field concentrates around the edges and in the gap between adjacent unit cells of the upper graphene layer (Figure 4a). This finding can be further confirmed by the power flow distribution shown in Figure 4e. Figure 4b shows weak surface currents on the VO_2_ film in the metallic phase, which are antiparallel to that of the upper graphene layer, and indicates weak magnetic resonance. Thus, the first resonance is caused by the coupling interaction between the neighboring unit cells, electric dipole resonance, and weak magnetic resonance.

At the second resonant frequency of 2.05 THz, the electric field is mainly localized around the edges of the individual graphene unit cells, as shown in Figure 4c. Moreover, power flow streams are observed across the center of the upper graphene unit cell (Figure 4f), indicating the excitation of the electric dipole resonance. Figure 4d shows that the surface currents on the VO_2_ film in the metallic phase are antiparallel to that of the upper graphene layer, forming a strong magnetic resonance. Electric dipole and magnetic resonances generally store electromagnetic energy and energy is dissipated due to the ohmic loss in graphene layer, thereby causing broadband absorption.

**Figure 5:**
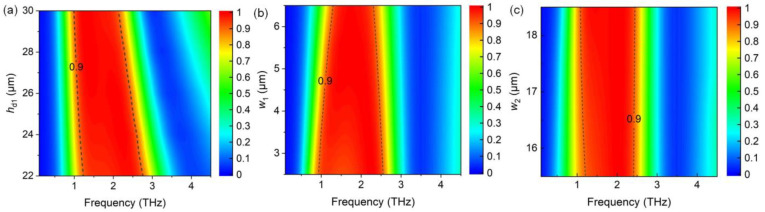
Varying absorption spectra with (**a**) the thickness of the Topas layer (*h*_d1_) between the lower graphene layer and VO_2_ film, and the distances from the unit boundary of the connected graphene squares (**b**) *w*_1_ and (**c**) *w*_2_, respectively.

In order to further investigate the absorption characteristics of the proposed absorber, absorption spectra are simulated with various geometrical parameters. Figure 5a shows that the absorption spectra vary with the thickness of the Topas layer (*h*_d1_) between the lower graphene layer and VO_2_ film. The first, and particularly second, resonances redshift due to the influence of the magnetic resonance between the upper graphene layer and VO_2_ film. Thus, the bandwidth of the broadband absorber increases as *h*_d1_ decreases. The amplitude attenuates slightly with increasing *h*_d1_, which can be explained well by the impedance matching with the free space. When *w*_1_ varied from 2.5 to 6.5 μm, the bandwidth decreased (Figure 5b). The first absorption resonance blue-shifts, which can be explained by the decreasing effective length of the LC circuit model, whereas the second resonant frequency shows a red-shift, which mainly resulted from the magnetic resonance. Figure 5c shows that the first resonance attenuated with decreasing *w*_2_, which can be explained by the impedance-matching theory.

**Figure 6:**
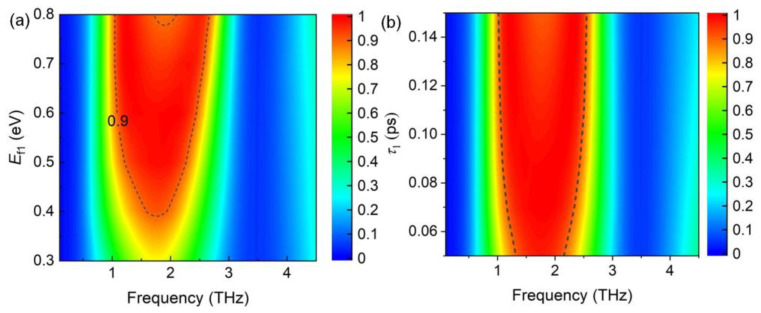
(**a**) Absorption spectra with increasing Fermi energy level *E*_f1_, and the carrier mobility μ is fixed as 1500 cm^2^V^−1^s^−1^. (**b**) Absorption spectra with varying relaxation time *τ*_1_, and the Fermi energy level is fixed as 0.6 eV.

Moreover, the bandwidth broadened with increasing Fermi energy level (Figure 6a). The bandwidth was ~1.35 THz at *E*_f_ = 0.7 eV, whereas the broadband almost disappeared when *E*_f_ = 0.3 eV. This indicates that the absorber can be used as an optical switch. Figure 6b shows the absorption spectra with varying relaxation times of graphene. It is clear that the relaxation time mainly influences the amplitude of the resonances, while showing a slight effect on the width of the absorption band.

### 3.2. Double Narrowband Absorption

**Figure 7:**
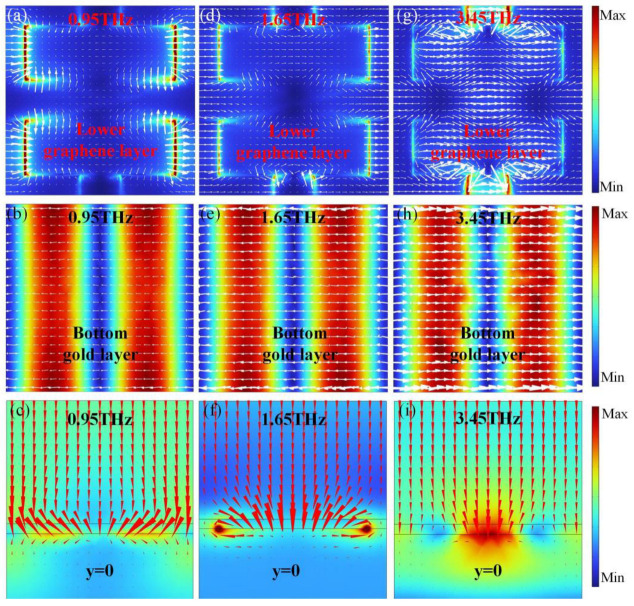
Distributions of the electric field amplitude |E| for (**a**,**d**,**g**) the upper graphene layer and (**b**,**e**,**h**) the bottom gold layer at the first, second, and third resonant frequencies of 0.95, 1.65, and 3.45 THz, respectively. Surface currents are marked with white arrows. Distributions of the power flow (red arrows) at the central cross-section of unit cell at (**c**) 1.45, (**f**) 1.65, and (**i**) 3.45THz, respectively.

The dual narrowband absorption mechanism is elucidated by investigating the electric field, surface current, and power flow distributions at different resonant frequencies, as shown in Figure 7. Figure 7a shows the distributions of electric field and surface currents in the lower graphene layer at the first resonant frequency of 0.95 THz with *E*_f2_ = 0.8 eV. The electric field was concentrated mainly around the ends and the gap of the square graphene unit cells, which originated from the electric dipole resonance and coupling between the adjacent unit cells. The distribution of surface currents in the upper graphene layer (Figure 7a) followed an opposite direction to that of the bottom gold layer (Figure 7b). Thus, weak magnetic resonance results in the first resonance.

Figure 7d shows the electric field and surface current distributions at the second resonant frequency of 1.65 THz. The electric field was concentrated mainly around the ends and edges of the square graphene unit cells, maintaining the electric dipole resonance. Figure 7e shows antiparallel surface currents in the bottom gold layer contrary to that in the lower graphene layer, which causes magnetic resonance. Thus, both the electric dipole resonance and magnetic resonance caused the second resonance. At the third resonant frequency of 3.45 THz, the electric field is mainly distributed in the ends of the graphene squares and two adjacent unit cells, as shown in Figure 7g. The presence of a strong magnetic resonance is indicated by the current distribution in Figure 7h. Thus, strong magnetic resonance and electric dipole resonance influenced the third resonance frequency. This finding is further confirmed by the power flow distributions at different resonant frequencies shown in Figure 7c,f,i.

**Figure 8:**
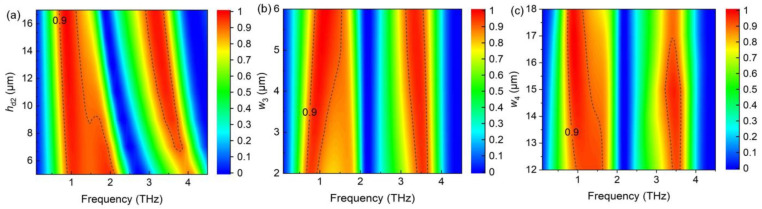
Absorption spectra varying with increasing (**a**) thickness of the Topas layer (*h*_d2_) between the VO_2_ film and bottom gold layer, and distances from the unit boundary of the connected graphene squares (**b**) *w*_3_ and (**c**) *w*_4_, respectively.

Figure 8 shows the dual narrowband absorption spectra with various parameters. As *h*_d2_ increases, the three resonant frequencies red-shift (Figure 8a) because the coupling strength of the magnetic resonance response is mainly determined by the thickness of the dielectric layer. As the magnetic resonance mainly affects the third resonant frequency, the resonance shifts at 3.45 THz are particularly evident. When *w*_3_ increases, the first resonance blue-shifts slightly due to the decreasing effective length of the LC circuit model, whereas the second and third resonances red-shifted due to magnetic resonance response (Figure 8b). As *w*_2_ increases, the absorption intensity of the first resonance increases slightly, whereas that of second and third resonances decrease, as shown in Figure 8c.

**Figure 9:**
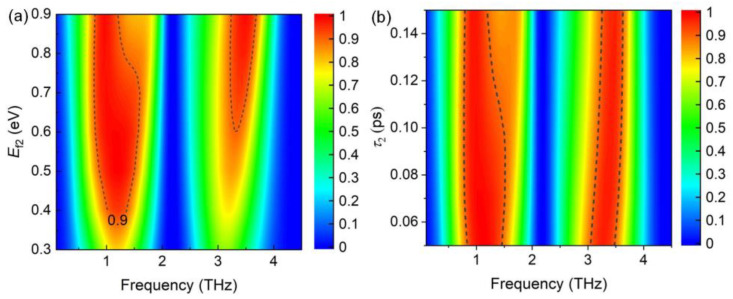
(**a**) Absorption spectra with increasing Fermi energy level *E*_f2_, and the carrier mobility μ is fixed as 1500 cm^2^V^−1^s^−1^. (**b**) Absorption spectra with varying relaxation time *τ*_2_, and the Fermi energy level is fixed as 0.8 eV.

As shown in Figure 9a, a double narrowband with absorption exceeding 90% can be achieved at an optimal *E*_f2_ value of 0.8 eV. The decreases of the Fermi energy affect the metallic performance of graphene; thus, the two absorption bands gradually disappeared. Figure 9b shows that the absorption intensity decreases with the increasing of relaxation times.

### 3.3. Influence of the Conductivity of VO_2_ Film

**Figure 10:**
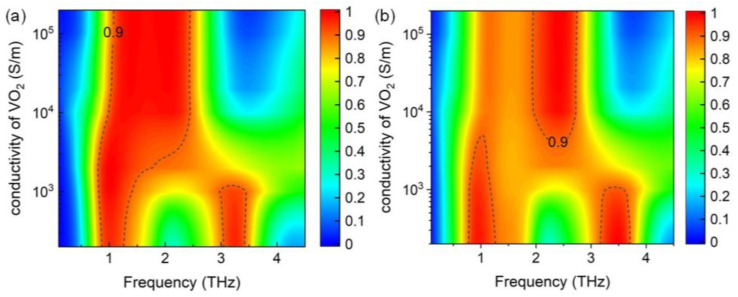
Absorption spectra of the proposed absorber with varying conductivity of the VO_2_ film, when (**a**) *E*_f1_ = 0.6 eV and *E*_f2_ = 0 eV; (**b**) *E*_f1_ = 0 eV and *E*_f2_ = 0.8 eV.

Figure 10 shows the influence of the conductivity of VO_2_ film on the absorption spectra. Broadband absorption is observed when the conductivity of the VO_2_ film is >5 × 10^3^ S/m, *E*_f1_ = 0.8 eV, and *E*_f2_ = 0 eV (Figure 10a). While the conductivity approaches zero, the VO_2_ film changes its state from metallic to insulating phase, corresponding to the variation from broadband absorption to dual narrowband absorption. This phenomenon can also be confirmed by the absorption spectrum shown in Figure 10b at *E*_f1_ = 0 eV and E_f2_ = 0.8 eV. Compared to Figure 10a, the Figure 10b shows a wider absorption bandwidth for the influence of the lower graphene layer, which contains larger unit cells and a shorter distance to the VO_2_ film. This phenomenon can be attributed to the increment of effective length and the decrement of the space of equivalent capacitance according to the LC circuit model.

**Figure 11:**
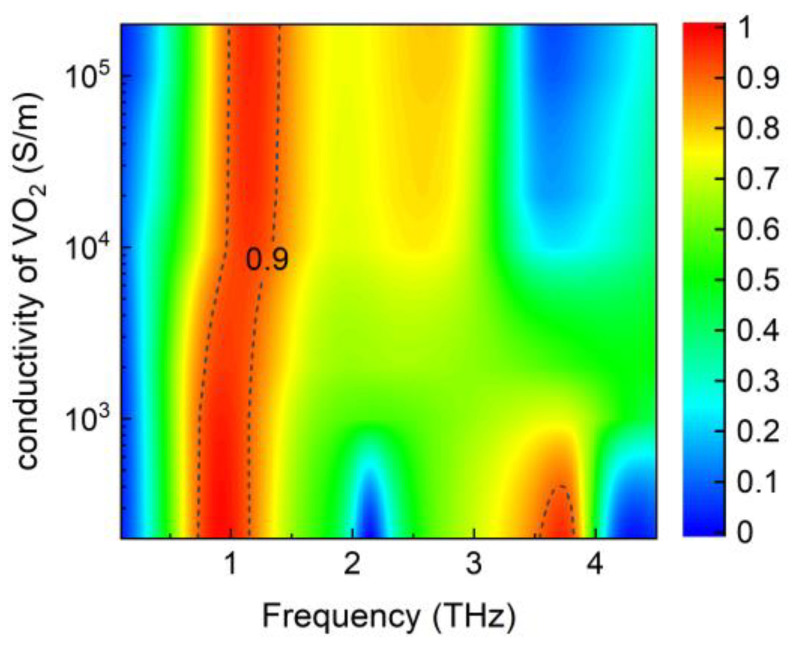
Absorption spectrum of the absorber with varying conductivity of the VO_2_ film at *E*_f1_ = *E*_f2_ = 0.8 eV.

**Figure 12:**
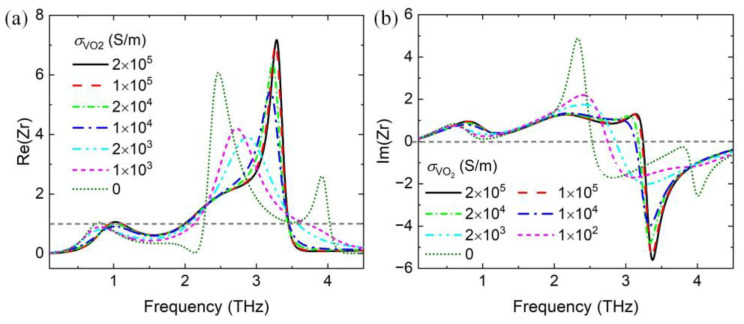
(**a**) Real parts and (**b**) imaginary parts of the relative impedance *Z*_r_ with different conductivities of VO_2_.

In addition, the multifunctional absorber can be further transformed into a single narrowband absorber by setting the conductivity of the VO_2_ film to appropriate values, as shown in Figure 11. Both the Fermi energy levels of the upper and lower graphene layers are set to 0.8 eV. A narrowband absorber can be realized at *σ*_VO2_ of >1000 S/m, particularly from 1000 to 10,000 S/m. This phenomenon can also be elucidated by the impedance-matching theory shown in Figure 12a,b.

The bottom gold layer prevents downward wave propagation as *σ*_VO2_ varies; thus, the transmittance calculated by |S_21_|^2^ is nearly zero. The absorptance and the relative impedance with normal incidence can be expressed as follows:

(6) $$A\left(\omega \right)=1-R\left(\omega \right)=1-\left|\frac{Z_{r}-1}{Z_{r}+1}\right|,$$

(7) $$Z_{r}=\sqrt{\frac{{\left(1+S_{11}\left(\omega \right)\right)}^{2}-S_{21}{\left(\omega \right)}^{2}}{{\left(1-S_{11}\left(\omega \right)\right)}^{2}-S_{21}{\left(\omega \right)}^{2}}},$$

where *Z*_r_ is the relative impedance between the proposed absorber and free space. Equation (5) shows that a perfect absorption can be achieved when the real and imaginary parts of *Z*_r_ approach 1 and 0, respectively. Figure 12a,b show that the impedances of the proposed absorber and free space are nearly matched in the frequency around 1 THz; these findings are consistent with the absorption spectra shown in Figure 11. Table 2 is the comparison of other multifunctional devices with the proposed absorber.

## 4. Conclusions

Herein, a dynamically switchable multifunctional absorber based on graphene metamaterials and VO_2_ film is numerically investigated in the THz regime. The absorber can serve as an either–or band selector due to the phase transition of VO_2_ film and the electrical controlled property of graphene. When the VO_2_ film is in the metallic phase and the Fermi energy levels of the upper and lower graphene layers are simultaneously set to 0 and 0.6 eV, respectively, broadband absorption with the absorptance of ~100% can be achieved. When the Fermi energy levels of the upper and lower graphene layers are set to 0.8 and 0 eV, and the VO_2_ film is in the insulating phase, a double narrowband can be realized with an excellent absorptance of >90%. Due to this flexible shift in above two functions, the proposed absorber realizes the optional bandwidth from 2.05 to 2.35 THz, and 3.25 to 3.6 THz, which denotes an either–or band selector application. Furthermore, a narrowband absorption can be obtained when the conductivity of VO_2_ is set as an appropriate value. The influence of varying geometrical parameters on the absorption spectra is also investigated, and field analyses are performed to understand the broadband absorption mechanism. Owing to these attractive properties, the proposed absorber can have promising applications such as multifunctional devices, switches, and band selectors.

## Figures and Tables

**Table 1:** Detailed dimensions of the proposed absorber.

Parameter	Description	Numerical Value
*P_x_*	Period of unit cell in the x-direction	38 μm
*P_y_*	Period of unit cell in the y-direction	38 μm
*h_d_*_0_	Spacer of the two graphene layers	2 μm
*h_d_*_1_	Thickness of the first Topas layer	26 μm
*h_d_*_2_	Thickness of the second Topas layer	11 μm
*h_VO_*_2_	Thickness of the VO_2_ layer	2 μm
*w*_1_	Distances from the unit boundary of the connected square graphene layers	4.5 μm
*w*_2_		18.5 μm
*w*_3_		4 μm
*w*_4_		15 μm

**Table 2:** Tunable multifunctional devices operating in the THz regime.

Reference	Functionality	Active Material	Tunning Method
[46]	Low-, high-, and multiband broadband	Graphene and VO_2_	Temperature and voltage
[47]	Multiband (six peaks) and broadband	Graphene and VO_2_	Temperature and voltage
[48]	Sensing and broadband	Si	Pump power
[49]	Broadband and narrowband	Graphene	voltage
[50]	Single narrowband and sensing	Graphene	Voltage
[51]	Dual-band and broadband	InSb and graphene	Temperature and voltage
[52]	Multiband (three peaks) and broadband	Graphene and VO_2_	Temperature and voltage
This study	Broadband and dual narrowband; single narrowband and either–or band selector	Graphene and VO_2_	Temperature and voltage

## Data Availability

Data are available in the main text.
